# Characteristics and prognosis of skip lymph node metastasis in gastric cancer: a retrospective study

**DOI:** 10.1186/s12957-025-03951-7

**Published:** 2025-07-24

**Authors:** Kuang-Hua Lo, Kuo-Hung Huang, Wen-Liang Fang, Shih-Chieh Lin, Yi-Ping Hung, Ming-Huang Chen, Chew-Wen Wu, Ching-Yun Kung

**Affiliations:** 1 Division of General Surgery, Department of Surgery, Taipei Veterans General Hospital, No. 201, Sec. 2, Shipai Rd., Beitou District, Taipei City, 11217 Taiwan; 2 School of Medicine, National Yang Ming Chiao Tung University, Taipei, Taiwan; 3 Department of Pathology, Taipei Veterans General Hospital, Taipei, Taiwan; 4 Center of Immuno-Oncology, Department of Oncology, Taipei Veterans General Hospital, Taipei, Taiwan

**Keywords:** Gastric cancer, Skip metastasis, Lymph node metastasis

## Abstract

**Background:**

Lymph node dissection is a standardized procedure in gastric cancer surgery. Typically, lymph node metastasis begins in the perigastric (PG) region and then extends to the extraperigastric (EP) region. However, in some circumstances, skip lymph node metastasis occurs in the EP region without involvement of the PG lymph nodes. This study aims to investigate the clinical significance of skip lymph node metastasis in gastric cancer.

**Methods:**

A total of 1,055 patients who underwent curative gastrectomy for primary gastric cancer with pathological lymph node metastasis were analyzed. Patients were categorized into three groups: the PG-only group, the PG + EP group, and the skip group. The clinicopathologic characteristics and prognosis were analyzed.

**Results:**

The incidence of skip lymph node metastasis was 3.9% (41 of 1,055 patients). The skip group had a higher proportion of females compared to both the PG-only group (43.9% vs. 27.5%, *p* = 0.025) and the PG + EP group (43.9% vs. 26.5%, *p* = 0.017). Additionally, the skip group showed a higher proportion of intestinal-type tumors compared to the PG-only group (68.3% vs. 50.6%, *p* = 0.029) and the PG + EP group (68.3% vs. 40.2%, *p* = 0.001). Disease-free survival and overall survival in the skip group were similar to those in the PG-only group but significantly better than those in the PG + EP group.

**Conclusions:**

Skip lymph node metastasis is uncommon, and it is associated with a higher proportion of females and intestinal-type tumors. The prognosis of the skip group was comparable to the PG-only group and significantly better than that of the PG + EP group.

## Introduction

Gastric cancer is the fourth leading cause of cancer-related deaths and the fifth most prevalent cancer worldwide [1]. Clinical and pathological classifications of gastric cancer are crucial for determining treatment strategies. Lymph node metastasis has been identified as a significant prognostic factor in gastric cancer. It influences treatment decisions, including standard gastrectomy with lymph node dissection, definitive chemotherapy, or adjuvant chemotherapy, and ultimately impacts patient prognosis [2].

Early gastric cancer is characterized by tumors confined to mucosa or submucosa (clinical T1), irrespective of lymph node involvement. Gastric cT1a tumors are associated with a low risk of lymph node metastasis, for which endoscopic treatment options such as endoscopic mucosal resection or endoscopic submucosal dissection are recommended. In contrast, gastric cT1b tumors demonstrate lymph node metastasis rates between 18% and 32%, and for these tumors, radical gastrectomy, including at least D1 lymph node dissection, is advised [3]. In patients with more advanced localized gastric cancer, radical distal or total gastrectomy is essential to achieve both adequate nodal dissection (at least D2 lymph node dissection) and microscopically negative (R0) margins [4, 5].

Furthermore, several studies have indicated that the location of lymph node metastasis influences overall survival [6]. Lymph nodes around the stomach are anatomically categorized and assigned specific station numbers. According to the Japanese gastric cancer classification, these lymph nodes are divided into the perigastric (PG) area (stations 1–6) and the extraperigastric (EP) area (stations 7–12) [7]. Theoretically, nodal metastasis typically originates in the peri-tumor region and subsequently spreads to the extraperigastric area. However, in clinical practice, metastasis to extraperigastric (EP) lymph nodes without perigastric (PG) involvement has been observed. This atypical metastatic pattern is referred to as skip lymph node metastasis. Although some previous studies have examined the prognosis and clinical significance of skip lymph node metastasis, no consensus has been reached [8–11], and the characteristics and prognosis of skip metastasis remain unclear, with limited published literature available. Therefore, this study aims to analyze the clinicopathological features of gastric cancer patients with lymph node metastasis to investigate the clinical significance of skip lymph node metastasis and its correlation with prognosis in gastric cancer.

## Materials and methods

### Patients

We reviewed data from 3,699 patients who underwent gastrectomy for primary gastric cancer at Taipei Veterans General Hospital between January 1990 and December 2020. All data were thoroughly reviewed, and each patient’s survival status was confirmed based on hospital records and the Taiwan National Health Insurance Research Database (NHIRD).

**Fig. 1:**
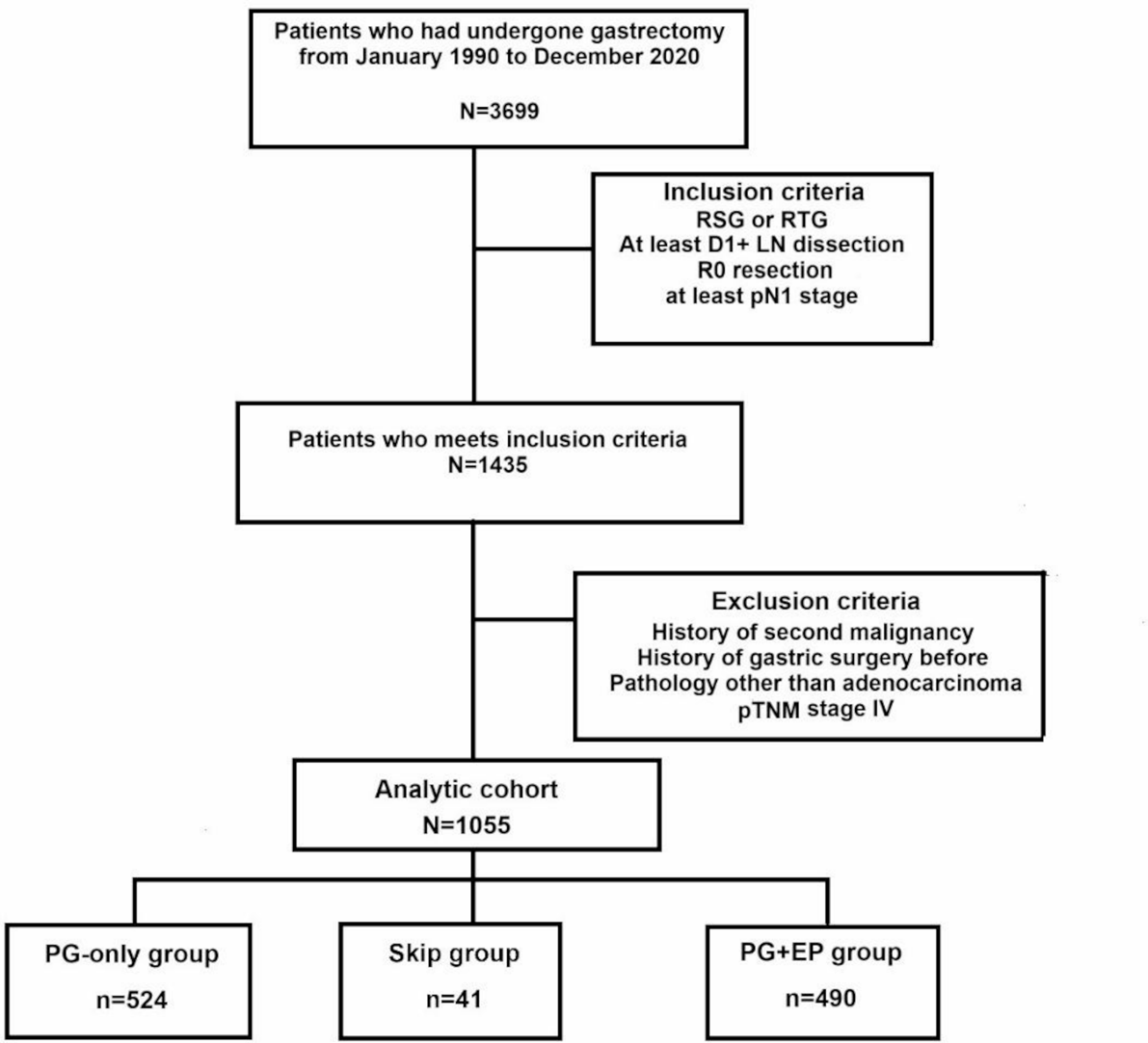
Flow chart of patient selection. RSG = radical subtotal gastrectomy, RTG = radical total gastrectomy, EP = extraperigastric, PG = perigastric, LN = lymph node

This study included patients who met the following criteria: (1) underwent upfront radical subtotal or total gastrectomy, (2) received at least D1 + lymph node dissection, (3) achieved R0 resection, and (4) had a minimum pN1 stage. Patients were excluded based on the following conditions: (1) presence of a second primary cancer, (2) previous gastric surgery, (3) pathology other than adenocarcinoma, and (4) stage IV disease. After applying these criteria, a total of 1,055 patients were selected for analysis (Fig. 1).

All patients underwent standardized preoperative evaluations prior to surgery. Radical subtotal gastrectomy was performed for tumors located in the middle or distal third of the stomach, while radical total gastrectomy was performed for tumors in the proximal third, infiltrative lesions (e.g., linitis plastica), or when the proximal margin was of concern during the operation.

For early gastric cancer, at least D1 + lymph node dissection was performed, while D2 lymph node dissection was carried out in cases of advanced gastric cancer. The gross pathological features of the specimens were assessed in terms of tumor location, size, and Borrmann classification. Microscopic features, including cell morphology, tumor infiltrative (INF) pattern (INF-α, INF-β, or INF-γ), tumor differentiation, Lauren classification (intestinal or diffuse type), Ming classification (expanding or infiltrating type), and lymphatic invasion, were also evaluated and analyzed [7].

Pathological staging was assigned in accordance with the 8th edition of the AJCC TNM classification system for gastric cancer [12]. Postoperative monitoring was conducted following institutional guidelines, with follow-up visits scheduled every 3 months during the first 3 years, and every 6 months thereafter until the patient’s death. These visits included physical examinations, laboratory tests for liver function panels, tumor markers (such as carbohydrate antigen 19 − 9 and carcinoembryonic antigen), chest X-rays, abdominal ultrasounds, and CT scans. If biopsies were not feasible, imaging studies were utilized to confirm the presence of tumor recurrence. The recurrence was categorized as locoregional, peritoneal, hematogenous, or distant lymphatic. Locoregional recurrence was identified in the remnant stomach, hepatoduodenal ligament, celiac axis, or peripancreatic area, while distant lymphatic recurrence included metastasis to para-aortic, Virchow’s, mediastinal, or inguinal lymph nodes, as well as lymphangitic spread to the lungs.

### Quality control

Taipei Veterans General Hospital is a high-volume tertiary medical center in Taiwan, with a specialized gastric cancer surgical team performing an average of 120 gastric cancer surgeries annually. All surgical procedures and pathological assessments were standardized according to the guidelines of the Japanese Gastric Cancer Association (JGCA). These standardized protocols have been consistently maintained across successive generations of surgeons, ensuring procedural uniformity over time. Immediately following resection, lymph node grouping was performed by the operator. Each surgeon had an equal opportunity to perform D1 or D2 lymph node dissection, minimizing operator-related bias. Regular case reviews and multidisciplinary conferences involving gastroenterologists, radiologists, pathologists, and surgeons ensured accurate and consistent pathological staging.

### Definition of the skip group

According to the Japanese gastric cancer classification, the lymph nodes surrounding the stomach are categorized into the perigastric (PG) area (stations 1–6) and the extraperigastric (EP) area (stations 7–12) [7]. Skip lymph node metastasis refers to the occurrence of metastasis in the EP region without involvement of the PG region. Based on this, patients were divided into three groups: the PG-only group (*n* = 524), the PG + EP group (*n* = 490), and the skip metastasis group (*n* = 41) (Fig. 1).

### Statistical analysis

To compare the clinicopathological characteristics across the three groups (PG-only, PG + EP, and skip), we applied appropriate statistical tests. For categorical data, we employed the chi-square test or Fisher’s exact test, while continuous data were analyzed using the Student’s t-test. The survival outcomes, including overall survival (OS) and disease-free survival (DFS), were assessed. OS was defined as death from any cause, while DFS referred to either recurrence or death from any cause, whichever occurred first following surgery. To assess survival outcomes across subgroups, we applied the Kaplan–Meier method for estimation and used the log-rank test to determine statistical significance. In an effort to control selection bias, we performed propensity score matching, thereby allowing for a more robust evaluation of prognostic differences between the skip group and the other groups. We constructed Cox regression models to evaluate the prognostic impact of skip lymph node metastasis, carefully adjusting for clinical and pathological variables. We also performed multivariate analyses to reduce confounding effects. Considering the evolution of clinical practices over time, we stratified survival analyses by treatment era. Statistical analyses were conducted using SPSS (v19.0; IBM Corp.). We regarded two-tailed *p*-values below 0.05 as statistically significant.

## Results

Among the 1,055 patients with positive nodal metastasis who underwent upfront curative standard gastrectomy for primary gastric cancer between January 1990 and December 2020, 524 patients (49.7%) had metastatic lymph nodes (LNs) confined to the perigastric area (PG-only group), while 490 patients (46.4%) had metastatic LNs in both the perigastric and extraperigastric areas (PG + EP group). The incidence of skip lymph node metastasis was 3.9% (41 out of 1,055 patients) among those with metastatic LNs.

The clinicopathological characteristics of the PG-only, skip and PG + EP groups are shown in Table 1. The univariate analysis showed that the skip group had a higher proportion of females in sex compared to both the PG-only group (43.9% vs. 27.5%, *p* = 0.025) and the PG + EP group (43.9% vs. 26.5%, *p* = 0.017). The skip group also showed a higher proportion of intestinal-type tumors in Lauren’s histology compared to the PG-only group (68.3% vs. 50.6%, *p* = 0.029) and the PG + EP group (68.3% vs. 40.2%, *p* = 0.001). Furthermore, the pN and pTNM stages in the skip group were lower than those in the PG-only and PG + EP groups. On the other hand, tumor size, Borrmann type, tumor cell infiltration, cell differentiation, Ming’s classification, and lymphatic invasion in the skip group were similar to those in the PG-only group. However, compared to the PG + EP group, the skip group had significantly smaller tumor sizes (5.3 ± 1.8 vs. 6.9 ± 3.2, *p* < 0.001), fewer cases of Borrmann type 3 and 4 (44.0% vs. 72.7%, *p* = 0.001), a higher proportion of expanding Ming’s classification (29.3% vs. 10.2%, *p* = 0.001), a higher proportion of alpha infiltration (14.6% vs. 6.1%, *p* = 0.031), and a higher proportion of differentiated tumor types (46.3% vs. 29.6%, *p* = 0.026). The number of retrieved lymph nodes (LNs) in the skip group was lower than in the PG-only group, but this difference was not statistically significant (33.1 ± 12.5 vs. 36.4 ± 14.8, *p* = 0.160). However, the number of retrieved LNs was significantly lower in the skip group compared to the PG + EP group (33.1 ± 12.5 vs. 40.0 ± 15.0, *p* = 0.004). The proportion of patients who received adjuvant chemotherapy did not differ significantly among the three groups.

**Table 1:** Baseline characteristics of the overall patient population before matching

	Skip group (*n* = 41)	PG-only group (*n* = 524)	*p* value (Skip vs. PG-only)	PG + EP group (*n* = 490)	*p* value (Skip vs. PG + EP)
Age(years)	66.3 ± 12.0	66.2 ± 12.3	0.944	65.7 ± 12.8	0.778
Sex			0.025*		0.017*
Male	23 (56.1%)	380 (72.5%)		360 (73.5%)	
Female	18 (43.9%)	144 (27.5%)		130 (26.5%)	
Tumor size(cm)	5.3 ± 1.8	5.4 ± 2.4	0.606	6.9 ± 3.2	< 0.001*
Tumor location			0.038*		0.108
Upper stomach	5 (12.2%)	53 (10.1%)		54 (11.0%)	
Middle stomach	22 (53.7%)	212 (40.5%)		174 (35.5%)	
Lower stomach	12 (29.3%)	252 (48.1%)		223 (45.5%)	
Whole stomach	2 (4.9%)	7 (1.3%)		39 (8.0%)	
Surgical method			0.363		0.558
Subtotal gastrectomy	30 (73.2%)	415 (79.2%)		337 (68.8%)	
Total gastrectomy	11 (26.8%)	109 (20.8%)		153 (31.2%)	
Infiltration			0.264		0.031*
α	6 (14.6%)	39 (7.4%)		30 (6.1%)	
β	16 (39.0%)	228 (43.5%)		149 (30.4%)	
γ	19 (46.3%)	257 (49.0%)		311 (63.5%)	
Gross appearance			0.472		0.001*
Superficial type	9 (22.0%)	106 (20.2%)		48 (9.8%)	
Borrmann type 1& 2	14 (34.1%)	139 (26.5%)		86 (17.6%)	
Borrmann type 3& 4	18 (43.9%)	279 (53.2%)		356 (72.7%)	
Lauren’s histology			0.029*		0.001*
Intestinal type	28 (68.3%)	265 (50.6%)		197 (40.2%)	
Diffuse type	13 (31.7%)	259 (49.4%)		293 (59.8%)	
Histology			0.403		0.026*
Undifferentiated	22 (53.7%)	316 (60.3%)		345 (70.4%)	
Differentiated	19 (46.3%)	208 (39.7%)		145 (29.6%)	
Ming’s Classification^a^			0.063		0.001*
expanding	12 (29.3%)	92 (17.6%)		50 (10.2%)	
infiltrating	29 (70.7%)	431 (82.3%)		440 (89.8%)	
Lymphatic invasion			0.562		0.007*
-	10 (24.4%)	150 (28.6%)		47 (9.6%)	
+	31 (75.6%)	374 (71.4%)		443 (90.4%)	
pT stage^b^			0.289		< 0.001*
pT1	9 (22.0%)	71 (13.5%)		22 (4.5%)	
pT2	6 (14.6%)	97 (18.5%)		38 (7.8%)	
pT3	11 (26.8%)	195 (37.2%)		178 (36.3%)	
pT4	15 (36.6%)	161 (30.7%)		252 (51.4%)	
pN stage^b^			< 0.001*		< 0.001*
pN1	35 (85.4%)	231 (44.1%)		15 (3.1%)	
pN2	5 (12.2%)	193 (36.8%)		115 (23.5%)	
pN3	1 (2.4%)	100 (19.1%)		360 (73.5%)	
pTNM stage^b^			0.037*		< 0.001*
I	8 (19.5%)	46 (8.8%)		4 (0.8%)	
II	17 (41.5%)	189 (36.1%)		40 (8.2%)	
III	16 (39.0%)	289 (55.2%)		446 (91.0%)	
Positive LNs	1.7 ± 1.4	4.8 ± 4.6	< 0.001*	16.0 ± 10.2	< 0.001*
Retrieved LNs	33.1 ± 12.5	36.4 ± 14.8	0.160	40.0 ± 15.0	0.004*
Adjuvant chemotherapy	7 (17.1%)	111 (21.2%)	0.533	128 (26.1%)	0.201
5 year OS	67.7%	58.7%	0.251	25.7%	< 0.001*
5 year DFS	60.1%	53.9%	0.251	21.6%	< 0.001*
Recurrence pattern					
Locoregional recurrence	3 (7.3%)	55 (10.5%)	0.788	88 (18.0%)	0.081
Peritoneal dissemination	7 (17.1%)	68 (13.0%)	0.463	114 (23.3%)	0.357
Hematogenous	6 (14.6%)	70 (13.4%)	0.825	94 (19.2%)	0.467
Distant lymphatic	1 (2.4%)	27 (5.2%)	0.712	80 (16.3%)	0.017*

Each *p* value was from a comparison with only the skip group before matching

Significant *p* values marked by * and bold font

^a^One data unavailable in PG-only group

^b^According to the American Joint Committee on Cancer staging manual, 8th edition

EP = extraperigastric, PG = perigastric, LNs = lymph nodes, OS = Overall survival rate, DFS = disease free survival rate

**Fig. 2:**
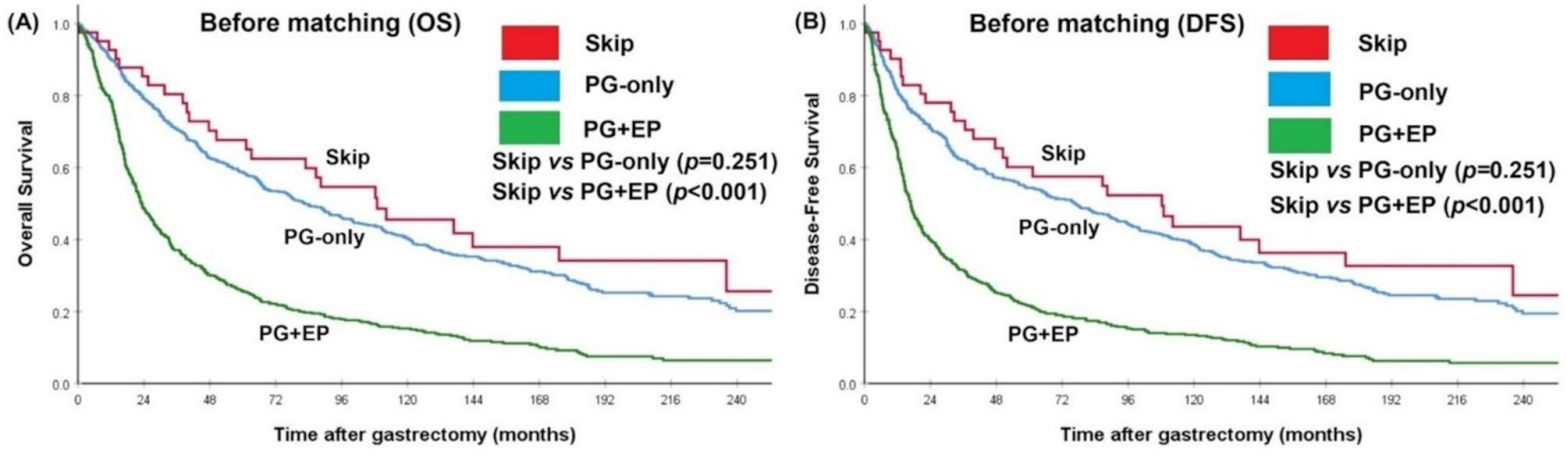
Overall survival (OS) and disease-free survival (DFS) of each group before matching. (**A**) OS of the skip group versus the PG-only group and PG + EP group, (**B**) DFS of the skip group versus the PG-only group and PG + EP group

The median follow-up duration was 68 months in the PG-only group, 89.4 months in the skip group, and 22.8 months in the PG + EP group. The overall survival (OS) rate and disease-free survival (DFS) rate in the skip group were similar to those in the PG-only group, but significantly better than those in the PG + EP group before matching (log-rank test, *p* < 0.001 for OS and *p* < 0.001 for DFS; Fig. 2A, B). The median OS with 95% CIs was 82.6 (68.3, 96.9) months in the PG-only group, 108.9 (57.3, 160.5) months in the skip group, and 23.3 (20.3, 26.2) months in the PG + EP group, respectively. The median DFS with 95% CIs was 76.6 (60.3, 93.0) months in the PG-only group, 108.3 (50.7, 165.9) months in the skip group, and 16.8 (14.7, 18.9) months in the PG + EP group, respectively. The recurrence pattern showed no significant difference between the skip and PG-only groups. However, the distant lymphatic recurrence rate in the PG + EP group was significantly higher than that in the skip group (16.3% vs. 2.4%, *p* = 0.017).

**Fig. 3:**
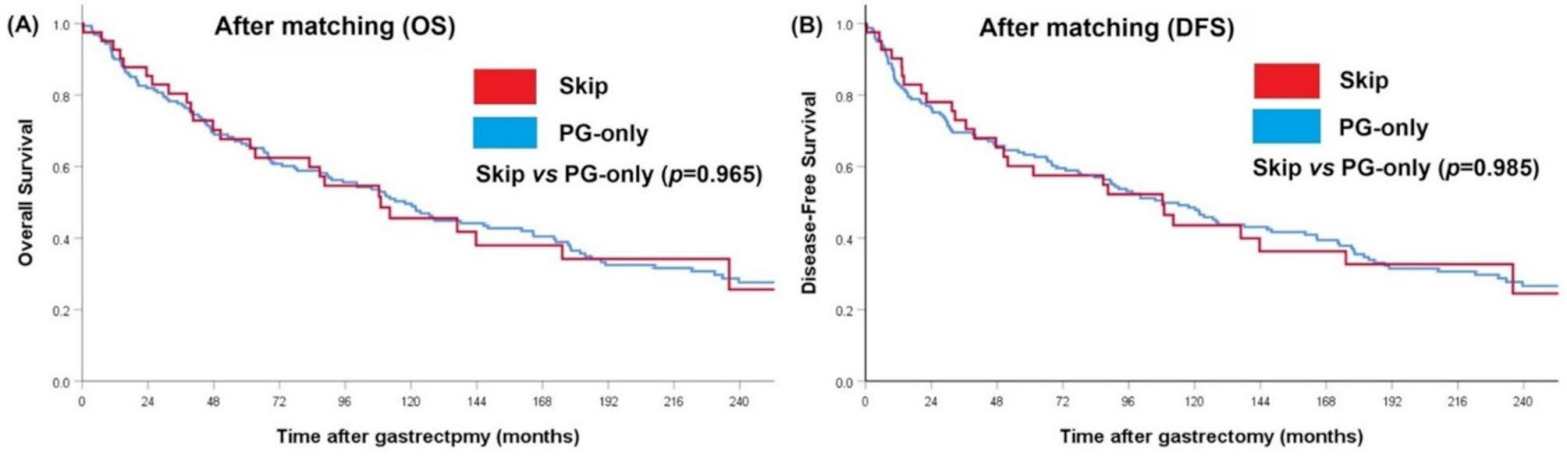
Overall survival (OS) and disease-free survival (DFS) of the skip group and the PG-only group after matching. (**A**) OS of the skip group versus the PG-only group, (**B**) DFS of the skip group versus the PG-only group

A 1:4 propensity score matching was conducted, using sex, Lauren’s histological subtype, and pTNM staging as matching factors, to control for potential confounding variables, in order to compare the survival of the skip group and that of the PG-only group. After matching, the baseline characteristics for both groups were found to be comparable with respect to the aforementioned factors (Table 2). The mean number of retrieved lymph nodes in the skip group was similar to that in the PG-only group (33.1 ± 12.5 vs. 34.8 ± 14.8, *p* = 0.505). Additionally, survival analysis revealed no significant difference between the skip and PG-only groups (log-rank test: *p* = 0.965 for overall survival (OS) and *p* = 0.985 for disease-free survival (DFS); Fig. 3A, B). The median OS with 95% CIs was 117.8 (93.4, 142.2) months in the PG-only group, and 108.9 (57.3, 160.5) months in the skip group. The median DFS with 95% CIs was 108.9 (80.5, 136.5) months in the PG-only group, and 108.3 (50.7, 165.9) months in the skip group. However, after matching, the skip group still had a higher proportion of upper & middle stomach with regard to tumor location (65.9% vs. 29.2%, *p* < 0.001), significantly higher proportion of total gastrectomy (26.8% vs. 3.1%, *p* < 0.001), lower pN stage (*p* = 0.016), fewer number of positive lymph node metastasis (1.7 ± 1.4 vs. 3.0 ± 2.9, *p* < 0.001), and a higher proportion of patients received adjuvant chemotherapy (17.1% vs. 1.9%, *p* = 0.001) compared to the PG-only group.

**Table 2:** Baseline characteristics of the overall patient population after matching

	Skip group (*n* = 41)	PG-only group (*n* = 161)	*p* value
Age(years)	66.3 ± 12.0	66.5 ± 11.7	0.909
Sex			0.791
Male	23 (56.1%)	94 (58.4%)	
Female	18 (43.9%)	67 (41.6%)	
Tumor size(cm)	5.3 ± 1.8	5.1 ± 2.1	0.700
Tumor location			< 0.001*
Upper stomach	5 (12.2%)	4 (2.5%)	
Middle stomach	22 (53.7%)	43 (26.7%)	
Lower stomach	12 (29.3%)	114 (70.8%)	
Whole stomach	2 (4.9%)	0 (0%)	
Surgical method			< 0.001*
Subtotal gastrectomy	30 (73.2%)	156 (96.9%)	
Total gastrectomy	11 (26.8%)	5 (3.1%)	
Infiltration			0.706
α	6 (14.6%)	18 (11.2%)	
β	16 (39.0%)	73 (45.3%)	
γ	19 (46.3%)	70 (43.5%)	
Gross appearance			0.371
Superficial type	9 (22.0%)	50 (31.1%)	
Borrmann type 1& 2	14 (34.1%)	40 (24.8%)	
Borrmann type 3& 4	18 (44.0%)	71 (44.1%)	
Lauren’s histology			0.942
Intestinal type	28 (68.3%)	109 (67.7%)	
Diffuse type	13 (31.7%)	52 (32.3%)	
Histology			0.378
Undifferentiated	22 (53.7%)	74 (46.0%)	
Differentiated	19 (46.3%)	87 (54.0%)	
Ming’s Classification			0.766
expanding	12 (29.3%)	51 (31.7%)	
infiltrating	29 (70.7%)	110 (68.3%)	
Lymphatic invasion			0.952
-	10 (24.4%)	40 (24.8%)	
+	31 (75.6%)	121 (75.2%)	
pT stage			0.808
pT1	9 (22.0%)	40 (24.8%)	
pT2	6 (14.6%)	32 (19.9%)	
pT3	11 (26.8%)	38 (23.6%)	
pT4	15 (36.6%)	51 (31.7%)	
pN stage			0.016*
pN1	35 (85.4%)	100 (62.1%)	
pN2	5 (12.2%)	45 (28.0%)	
pN3	1 (2.4%)	16 (9.9%)	
pTNM stage			0.933
I	8 (19.5%)	30 (18.6%)	
II	17 (41.5%)	63 (39.1%)	
III	16 (39.0%)	68 (42.2%)	
Positive LNs	1.7 ± 1.4	3.0 ± 2.9	< 0.001*
Retrieved LNs	33.1 ± 12.5	34.8 ± 14.8	0.505
Adjuvant chemotherapy	7 (17.1%)	3 (1.9%)	0.001*
5 year OS	67.7%	65.8%	0.965
5 year DFS	60.1%	63.3%	0.985
Recurrence pattern			
Locoregional recurrence	3 (7.3%)	21 (13.0%)	0.312
Peritoneal dissemination	7 (17.1%)	20 (12.4%)	0.435
Hematogenous	6 (14.6%)	18 (11.2%)	0.589
Distant lymphatic	1 (2.4%)	12 (7.5%)	0.474

Each *p* value was from a comparison with only the skip group after matching

Significant *p* values marked by * and bold font

PG = perigastric, LNs = lymph nodes, OS = Overall survival rate, DFS = disease free survival rate

Thus, we further conducted Cox proportional hazards model and multivariable analysis in order to test independent prognostic significance of skip lymph node metastasis after adjusting for potential confounders, and the results are shown in Table 3. After propensity score matching, univariate Cox regression analysis identified several clinicopathological factors significantly associated with overall survival (OS), including age > 65 years (HR: 3.23; 95% CI: 2.14–4.87; *p* < 0.001), male sex (HR: 1.60; 95% CI: 1.12–2.28; *p* = 0.01), larger tumor size (HR: 1.14; 95% CI: 1.05–1.90; *p* = 0.023), Borrmann type III/IV (HR: 1.44; 95% CI: 1.17–1.76; *p* < 0.001), Lauren diffuse type (HR: 1.92; 95% CI: 1.28–2.86; *p* = 0.001), undifferentiated histology (HR: 1.56; 95% CI: 1.10–2.21; *p* = 0.012), lymphatic invasion (HR: 1.56; 95% CI: 1.03–2.37; *p* = 0.037), and advanced pTNM stage (HR: 1.66; 95% CI: 1.30–2.10; *p* < 0.001). In the multivariate analysis, only three variables remained independently associated with poor overall survival, including age > 65 years (HR: 3.19; 95% CI: 2.11–4.84; *p* < 0.001), undifferentiated histology (HR: 1.44; 95% CI: 1.01–2.05; *p* = 0.042), and higher pTNM stage (HR: 1.79; 95% CI: 1.40–2.30; *p* < 0.001). Notably, lymph node status, comparing PG-only and skip metastasis, was not significantly associated with overall survival in either univariate (HR: 0.97; 95% CI: 0.64–1.53; *p* = 0.965) or multivariate analysis. Similarly, other factors such as tumor location, surgical method, infiltration pattern, Ming classification, and receipt of adjuvant chemotherapy did not show significant prognostic impact in the adjusted model.

**Table 3:** Univariate analysis and multivariate analysis for overall survival associated with gastric cancer patients after matching

Factors	Univariate analysis		Multivariate analysis	
	HR (95% CI)	*p* value	HR (95% CI)	*p* value
Age (> 65 vs. < 65)	3.23 (2.14, 4.87)	< 0.001*	3.19 (2.11, 4.84)	< 0.001*
Sex (male vs. female)	1.60 (1.12, 2.28)	0.01*		
Tumor size (large vs. small)	1.14 (1.05, 1.90)	0.023*		
Tumor location	1.25 (0.93, 1.68)	0.137		
Surgical method (subtotal vs. total)	1.24 (0.92, 1.67)	0.158		
Infiltration (γ, β vs. α)	0.89 (0.70, 1.14)	0.357		
Borrmann type (3&4 vs. 1&2)	1.44 (1.17, 1.76)	< 0.001*		
Lauren (Diffuse vs. Intestinal)	1.92 (1.28, 2.86)	0.001*		
Histology (Unifferentiated vs. Differentiated)	1.56 (1.10, 2.21)	0.012*	1.44 (1.01, 2.05)	0.042*
Ming (expanding vs. infiltrating)	0.87 (0.61, 1.23)	0.427		
Lymphatic invasion (Pos vs. Neg)	1.56 (1.03, 2.37)	0.037*		
pTNM stage (III->II->I)	1.66 (1.30, 2.10)	< 0.001*	1.79 (1.40, 2.30)	< 0.001*
Adjuvant chemotherapy (received vs. nil)	0.98 (0.40, 2.41)	0.970		
LN status (PG-only vs. Skip)	0.97 (0.64, 1.53)	0.965		

Values in parentheses are 95% confidence intervals

Significant p values marked by * and bold font

HR = hazard ratio, CI = confidence intervals, Pos = positive, Neg = negative

**Fig. 4:**
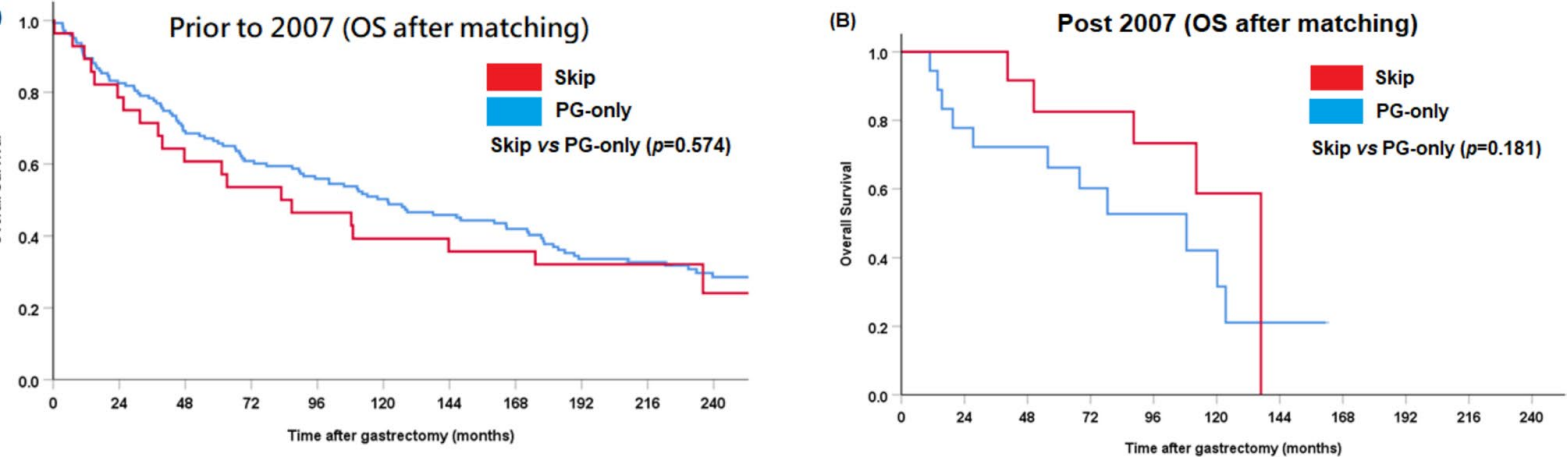
Overall survival (OS) of each status of lymph node metastasis with Kaplan–Meier curves after matching, stratified by prior to 2007 and post 2007. (**A**) Prior to 2007, (**B**) Post 2007

In addition, due to extended study period of over 30 years, we performed subgroup survival analysis stratified by treatment period to address era-based heterogeneity. For subgroup analysis, the year 2007 was selected as the cut-off point to stratify patients into two temporal cohorts (≤ 2007 vs. > 2007), based on Adjuvant Chemotherapy Trial of TS-1 for Gastric Cancer (ACTS-GC) — involving patients with stage II or III gastric cancer who underwent D2 surgery [13]. Survival analysis with Kaplan–Meier method revealed no significant difference between the skip and PG-only groups no matter prior to 2007 or post 2007 (log-rank test: *p* = 0.574 for ≤ 2007 group and *p* = 0.181 for > 2007 group; Fig. 4A, B).

The distribution of skip lymph node (LN) metastasis is summarized in Table 4. The most frequently affected site was station 7 (51.2%), followed by station 8a (34.1%) and station 11 (12.2%).

**Table 4:** The location of skip metastasis and the number of involved stations

Location of skip LNs	Number of patients(*n* = 41)
Station 7	21 (51.2%)
Station 8a	14 (34.1%)
Station 9	2 (4.9%)
Station 10	1 (2.4%)
Station 11	5 (12.2%)
Station 12a	1 (2.4%)
Station 13	1 (2.4%)
Station 14	1 (2.4%)

LN: lymph node

## Discussion

Gastric cancer generally exhibits a higher incidence in males than in females, with a male-to-female ratio of approximately 2:1. Previous studies have indicated that female sex is a favorable prognostic factor in gastric cancer [14, 15]. Several independent factors have been identified as associated with prognosis. Smaller tumor size, Borrmann type 1 & 2 lesions in gross appearance [16], differentiated histological types [17], INF alpha pattern [18, 19], expanding type according to Ming’s classification [20, 21], intestinal type based on Lauren’s histology [22, 23], and the absence of lymphatic invasion generally predict a better prognosis in gastric cancer. In this study, we observed that the skip group had a higher proportion of females and intestinal-type tumors in Lauren’s histology compared to both the PG-only and PG + EP groups. Moreover, the pN and pTNM stages of the skip group were lower than those of the PG-only and PG + EP groups. Additionally, compared to the PG + EP group, the skip group exhibited significantly smaller tumor sizes, fewer cases of Borrmann type 3 & 4, a higher proportion of expanding type lesions according to Ming’s classification, INF alpha infiltration, and a greater proportion of differentiated histological types.

Based on the characteristics mentioned above, it appeared that the prognosis of patients in the skip group would be better than that of patients in both the PG-only and PG + EP groups. Prior to matching, Kaplan–Meier survival curves for overall survival (OS) and disease-free survival (DFS) across different lymph node metastasis statuses suggested that the prognosis of the skip group was better compared to the PG-only group, although the difference was not statistically significant. The OS and DFS of the skip group were significantly better than those in the PG + EP group, which was reasonable. To eliminate the effects of confounding factors, we conducted propensity score matching based on characteristics such as pTNM stage, sex, and Lauren’s histology. After matching, survival curves for the skip and PG-only groups were nearly identical. Despite the application of propensity score matching, certain prognostic variables, including tumor location, surgical procedure, receipt of adjuvant chemotherapy, and notably pN stage remained significantly imbalanced between the skip and PG-only lymph node metastasis groups. To account for these discrepancies, we conducted Cox proportional hazards model and multivariable analyses to evaluate the independent significance of prognostic factors. However, lymph node status, comparing PG-only and skip metastasis, was not significantly associated with overall survival in either univariate or multivariate models. Likewise, other clinical factors such as tumor location, surgical approach, and administration of adjuvant chemotherapy did not exhibit significant prognostic value after adjustment. Although a discrepancy in pN stage distribution was observed between the groups, the pTNM stage provides a more comprehensive and integrated assessment of tumor burden and biological behavior. Importantly, no significant imbalance in pTNM stage was noted between the skip and PG-only groups after matching. The non-significant association between adjuvant chemotherapy and overall survival also warrants consideration. This may be attributed to the relatively low proportion of patients who received adjuvant treatment in our matched cohort, potentially introducing statistical imprecision or selection bias. Moreover, treatment adherence, influenced by patient frailty, toxicity, or early-stage disease, may have further diminished the observable benefit. Given the well-established survival advantage of adjuvant chemotherapy in stage II and III gastric cancer [13], our findings should be interpreted with caution.

In addition, due to extended study period of over 30 years, we performed subgroup survival analysis stratified by treatment period to address era-based heterogeneity. For subgroup analysis, the year 2007 was selected as the cut-off point to stratify patients into two temporal cohorts (≤ 2007 vs. > 2007), based on Adjuvant Chemotherapy Trial of TS-1 for Gastric Cancer (ACTS-GC), published in 2007 [13]. S-1 is an effective adjuvant treatment for East Asian patients who have undergone a D2 dissection for locally advanced gastric cancer (stage II (excluding T1 cases), IIIA, or IIIB). Survival analysis with Kaplan–Meier method revealed no significant difference between the skip and PG-only groups no matter prior to 2007 or post 2007. However, it is worth noting that, regardless of lymph node status, patients in the prior to 2007 subgroup exhibited numerically worse 5-year overall survival rates—65.7% vs. 66.2% (*p* = 0.35) in the PG-only group and 60.7% vs. 82.5% (*p* = 0.256) in the skip metastasis group, although these differences did not reach statistical significance. This trend may reflect improvements in evolving treatment guidelines in the modern era, despite the lack of formal significance in our matched cohort.

Disease-free survival and overall survival in the skip group were similar to those in the PG-only group but significantly better than those in the PG + EP group according to our results. These results are consistent with those reported in previous studies [8, 10, 24, 25]. H. Saito et al. [9] observed that patients with skip lymph node metastasis had a prognosis comparable to those with metastasis confined to the PG-only group, but better than those in the PG + EP group. However, contrasting findings have been reported in the other literature. Y. Y. Choi et al. [11] found that the skip metastasis group had worse survival than the PG-only group and a similar prognosis to the PG + EP group when adjusting for tumor stage. Notably, their study found that the number of lymph nodes retrieved in the skip group was significantly lower than in the other groups, which raised concerns about whether the observed poorer prognosis was due to the presence of skip metastasis or possibly the result of insufficient lymph node sampling. To evaluate the adequacy of lymph node dissection, we compared the number of lymph nodes retrieved among the skip, PG-only, and PG + EP groups. The counts for the skip and PG-only groups were similar both before and after matching, though the PG + EP group had a higher retrieval number. Nevertheless, the total number of lymph nodes retrieved in all groups was sufficient to confirm that the dissection process met the necessary standards. Furthermore, we analyzed the recurrence patterns in each group to assess whether skip lymph node metastasis is associated with a higher risk of distant lymphatic recurrence. The distant lymphatic recurrence rate showed no significant difference between the skip and PG-only groups but was significantly lower than in the PG + EP group. Given that over 70% of patients in the PG + EP group had pN3 disease, indicating a significantly higher nodal metastatic burden, this finding is understandable. Therefore, we concluded that there was no significant difference in survival rate or the distant lymphatic recurrence rate between patients with skip lymph node metastasis and those with perigastric-only nodal involvement after matching.

Furthermore, this finding raises an important question: is the number of metastatic lymph nodes (LNs) more significant than their location? Some studies have shown that the location of positive nodes does not significantly affect median survival when adjusted for the number of positive nodes. In contrast, the number of positive lymph nodes have a profound impact on survival [26]. However, other studies suggest that the anatomical extent of metastatic LNs remains an important prognostic factor in gastric cancer [6, 27]. The definition of pN stage has evolved, from the UICC 1987 4th edition, which classified metastatic LNs by location, to the UICC 1997 5th edition, which classified them by number of metastatic nodes (Table 5).

**Table 5:** The 1987 UICC, 1997 UICC and 2016 AJCC TNM classifications for lymph node staging

*N* stage	UICC 1987 4th edition	UICC 1997 5th edition	AJCC 2016 8th edition
N0	No regional lymph node metastasis	Histological examination of a regional lymphadenectomy specimen will ordinarily include ≥ 15 lymph nodes	No regional lymph node metastasis
N1	Metastasis in perigastric lymph node(s) ≤ 3 cm of the edge of the primary tumor	1–6 regional lymph node metastases	1–2 regional lymph nodes metastasis
N2	Metastasis in perigastric lymph node(s) > 3 cm from the edge of the primary tumor or in lymph nodes along the left gastric, common hepatic, splenic or celiac arteries	7–15 regional lymph node metastases	3–6 regional lymph nodes metastasis
N3	-	≥ 16 regional lymph node metastases	N3a: 7–15 regional lymph nodes metastasis
			N3b: ≥ 16 regional lymph node metastases
M1	Metastasis in hepatoduodenal, retropancreatic, mesenteric or para-aortic nodes	Metastasis in retropancreatic, mesenteric or para-aortic nodes	-

UICC: Union Internationale contre le Cancer (Hermanek and Sobin, 1987; Sobin and Wittekind, 1997)

AJCC: American Joint Committee on Cancer

Indeed, the 5th edition of the TNM classification provides a more accurate estimation of prognosis [28, 29]. Nonetheless, a recent large-scale study highlighted the potential significance of topographic information regarding specific nodal stations [30, 31]. M.E. Sayegh et al. [32] discussed the advantages and disadvantages of the number-based N-staging system (UICC) versus the anatomical-based N-staging system (JGCA, Japanese Gastric Cancer Association). The JGCA system is highly detailed and anatomically based, offering essential guidance for surgical treatment, which is its primary focus. In contrast, the UICC system provides no direct treatment guidance, but it is primarily used to accurately assess the metastatic burden and, consequently, prognosis. While our results support the prognostic reliability of the current UICC/AJCC (American Joint Committee on Cancer) TNM staging system, which evaluates lymph node status based on the number of positive nodes, the extent of lymph node dissection in gastric cancer surgery should adhere to JGCA guidelines. Our findings indicate that the majority of skip nodal metastases occur at stations 7 (51.2%), 8a (34.1%), and 11 (12.2%). Based on this distribution, gastrectomy with at least D1 lymph node dissection should be considered the minimum standard, as station 7 is the most common site for skip metastases. If the risk of skip nodal metastasis is considered, D2 dissection should be routinely performed whenever feasible.

In addition, there were several studies to discuss whether sentinel node (SN) technique and sentinel node navigation surgery (SNNS) are feasible or not in gastric cancer in order to reduce morbidity of extensive lymph node dissection. A prospective multicenter trial in Japan [33] revealed that endoscopic dual tracer method for SN biopsy was feasible when applied to the superficial, relatively small gastric adenocarcinomas. The Sentinel Node ORIented Tailored Approach (SENORITA) trial [34] also demonstrated that there was no significant difference about 5-year OS and DFS between laparoscopic sentinel node navigation surgery group and conventional standard gastrectomy plus LN dissection. Nevertheless, the incidence of metachronous gastric cancer in the laparoscopic sentinel node navigation surgery group was still significantly higher. Skip lymph node metastasis may be one of the potential factors contributing to this phenomenon. Skip lymph node metastasis undermines the fundamental assumption of sentinel lymph node (SLN) theory, which posits that tumor cells first reach specific “sentinel” nodes before disseminating to others. Several mechanistic explanations have been proposed to account for skip metastases [35]. Anatomical variability in gastric lymphatic drainage is one of the most cited causes. The stomach has a highly complex and variable lymphatic system, with multiple drainage pathways that allow tumor cells from specific regions, such as the lesser curvature or antrum, to bypass expected nodal stations and reach more distal basins, including stations No. 7, 8a, or 12a. Lymphatic mapping studies using indocyanine green (ICG) fluorescence or radiotracers have supported the presence of such non-linear drainage routes. Also, lymphatic obstruction caused by peritumoral fibrosis or tumor emboli may redirect lymph flow toward alternate, often more distant, nodal pathways. From a surgical standpoint, the presence of skip metastases presents a critical limitation to SLN navigation surgery in gastric cancer. While SLN mapping has shown promise in early gastric cancer, the possibility of false-negative results due to undetected skip metastasis raises concerns about oncologic safety. SLN-based limited lymph node dissection may result in understaging and inadequate resection. In the future, we hope to further utilize the characteristic patterns observed in patients with skip lymph node metastasis to develop predictive models. Then integrating this predictive model, sentinel lymph node (SLN) mapping with preoperative imaging techniques, and intraoperative navigation technologies may facilitate the development of a hybrid surgical approach. This strategy, which incorporates selective extended lymph node dissection based on individualized risk assessment for skip lymph node metastasis, may offer an optimal balance between oncologic curability and surgical preservation.

To the best of our knowledge, this is the largest cohort study investigating skip lymph node metastasis in gastric cancer conducted outside Japan and South Korea. Still, there are several limitations in our study. First, it is a single-center, retrospective study that includes only a Taiwanese population, which may introduce potential selection bias. Second, this cohort study spans a period of 30 years, during which treatment guidelines have evolved. Third, the number of patients in the skip metastasis group is limited due to the low incidence of skip lymph node metastasis (3.9%), but the Power (1-β) = 0.8117953, which is acceptable. Fourth, it is also possible that minor perigastric metastases were missed intraoperatively or during pathological examination. Nevertheless, we made every effort to minimize potential sources of bias. All surgical procedures and pathological classifications were performed by the same team of high-volume gastric surgeons and gastrointestinal pathologists throughout the study period, enhancing procedural uniformity. In addition, we conducted subgroup analyses stratified by treatment era to account for possible changes in clinical practice over time. While we acknowledge the inherent limitations of a retrospective study, we believe our findings offer meaningful insights into the clinicopathological characteristics of skip lymph node metastasis and its potential prognostic relevance in gastric cancer.

## Conclusion

Skip lymph node metastasis is uncommon, with an incidence of 3.9%. It is associated with a higher proportion of females and intestinal-type tumors compared to both the PG-only group and the PG + EP group. The overall survival rate and disease-free survival rate in the skip group are similar to those in the PG-only group, but significantly better than those in the PG + EP group.

## Data Availability

No datasets were generated or analysed during the current study.
